# Exploring the role of Nrf2 signaling in glioblastoma multiforme

**DOI:** 10.1007/s12672-022-00556-4

**Published:** 2022-09-28

**Authors:** Wireko Andrew Awuah, Abdul-Rahman Toufik, Rohan Yarlagadda, Tatiana Mikhailova, Aashna Mehta, Helen Huang, Mrinmoy Kundu, Leilani Lopes, Sylvester Benson, Lyndin Mykola, Sikora Vladyslav, Athanasios Alexiou, Badrah S. Alghamdi, Anwar M. Hashem, Ghulam Md Ashraf

**Affiliations:** 1 Sumy State University, Sumy, Ukraine; 2 Rowan University School of Osteopathic Medicine, Stratford, NJ USA; 3 SUNY Upstate Medical University, Syracuse, NY 13210 USA; 4 University of Debrecen-Faculty of Medicine, Debrecen, 4032 Hungary; 5 Royal College of Surgeons in Ireland, University of Medicine and Health Sciences, Dublin, Ireland; 6 Institute of Medical Sciences and SUM Hospital, Bhubaneswar, India; 7 College of Osteopathic Medicine of the Pacific-Northwest, Western University of Health Sciences, Lebanon, OR USA; 8 Columbia University, New York, USA; 9 Department of Science and Engineering, Novel Global Community Educational Foundation, Hebersham, NSW 2770 Australia; 10 AFNP Med, 1030 Vienna, Austria; 11 Department of Physiology, Neuroscience Unit, Faculty of Medicine, King Abdulaziz University, Jeddah, Saudi Arabia; 12 Pre-Clinical Research Unit, King Fahd Medical Research Center, King Abdulaziz University, Jeddah, Saudi Arabia; 13 Department of Medical Microbiology and Parasitology, Faculty of Medicine, King Abdulaziz University, Jeddah, 21589 Saudi Arabia; 14 Vaccines and Immunotherapy Unit, King Fahd Medical Research Center, King Abdulaziz University, Jeddah, 21589 Saudi Arabia; 15 Department of Medical Laboratory Sciences, Faculty of Applied Medical Sciences, King Abdulaziz University, Jeddah, 21589 Saudi Arabia

**Keywords:** Glioblastoma, Nrf-2 expression, Temozolomide, Molecular signaling pathways, MAP/ERK, JAK–STAT, c-Myc

## Abstract

Glioblastoma multiforme (GBM) is one of the most aggressive glial cell tumors in adults. Although current treatment options for GBM offer some therapeutic benefit, median survival remains poor and does not generally exceed 14 months. Several genes, such as isocitrate dehydrogenase (IDH) enzyme and *O*6-methylguanine-DNA methyltransferase (MGMT), have been implicated in pathogenesis of the disease. Treatment is often adapted based on the presence of IDH mutations and MGMT promoter methylation status. Recent GBM cell line studies have associated Nuclear Factor Erythroid 2-Related Factor 2 (Nrf2) expression with high-grade tumors. Increased Nrf2 expression is often found in tumors with IDH-1 mutations. Nrf2 is an important transcription factor with anti-apoptotic, antioxidative, anti-inflammatory, and proliferative properties due to its complex interactions with multiple regulatory pathways. In addition, evidence suggests that Nrf2 promotes  GBM cell survival in hypoxic environment,by up-regulating hypoxia-inducible factor-1α (HIF-1α) and vascular endothelial growth factor (VEGF). Downregulation of Nrf2 has been shown to improve GBM sensitivity to chemotherapy drugs such as Temozolomide. Thus, Nrf2 could be a key regulator of GBM pathways and potential therapeutic target.  Further research efforts exploring an interplay between Nrf2 and major molecular signaling mechanisms could offer novel GBM drug candidates with a potential to significantly improve patients prognosis.

## Introduction

**Fig. 1:**
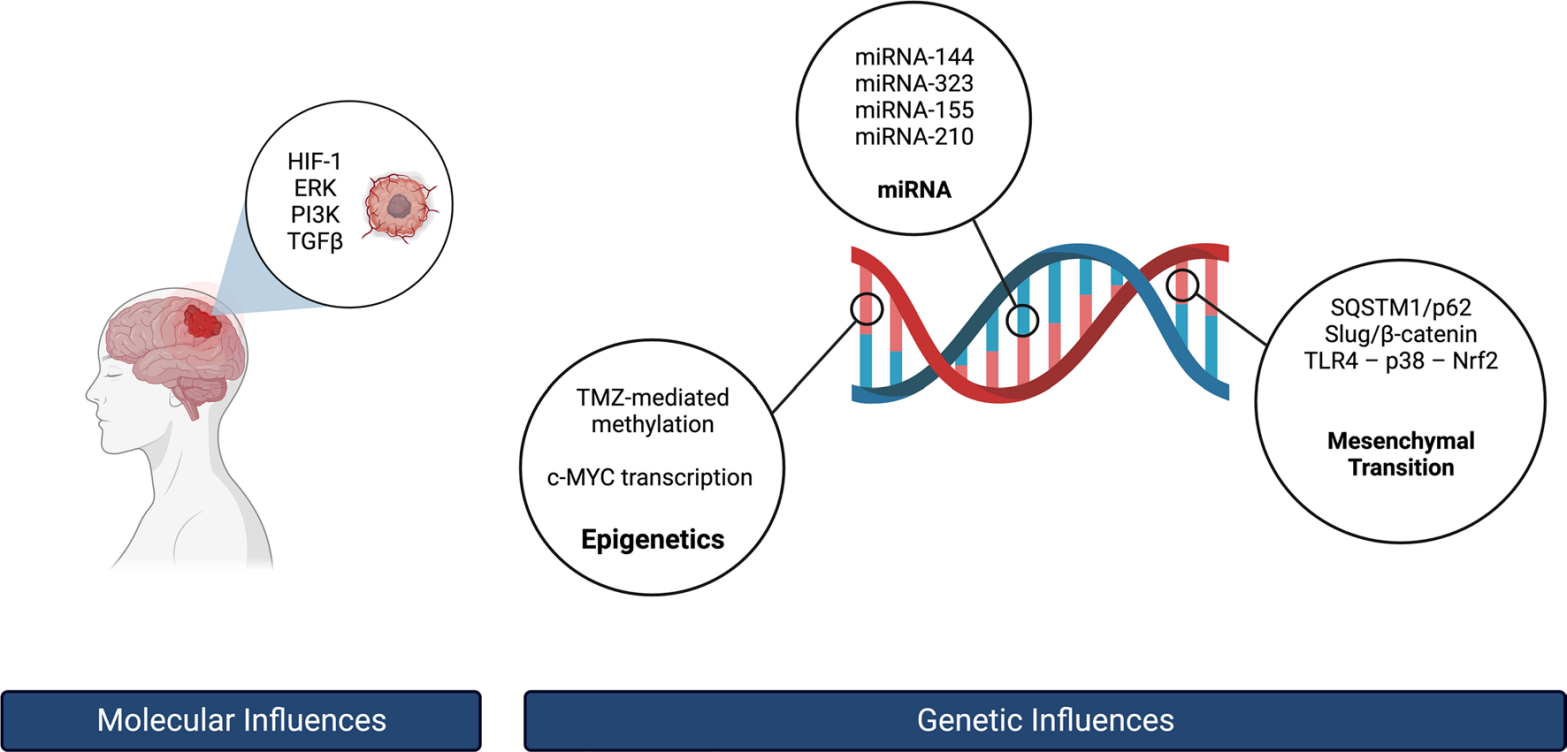
An overview of the molecular and genetic influences of Nrf2 on GBM formation. (Created with BioRender.com)

Glioblastoma multiforme (GBM) is a glial cell tumor known for its aggressive nature, accounting  for approximately 45.2% of primary malignant brain tumors of the CNS [1]. Despite emerging GBM treatments, the median survival of patients is reported to be around 14 months [2]. Total surgical resection is recommended early after the diagnosis to improve patient outcomes [3]. In most cases, GBM is found in the form of a localized tumor; metastases,  though uncommon, can  occur [4]. The development of GBM and other malignant gliomas have been associated with radiation exposure and genetic changes, however, its etiology is believed to be complex and caused by dysregulation of various molecular mechanisms, as shown in Fig. 1 [5, 6]. While different genetic and epigenetic alterations are implicated in GBM, identifying key disease mechanisms responsible for treatment-resistant phenotype remains a challenge. [1]. The name *multiforme* is itself a reference to  the high degree of genetic variability in these tumors [7]. Describing tumor gene expression profiles has become essential to develop clinically relevant classifications and appropriate therapeutic strategies [6]. The status of isocitrate dehydrogenase (IDH) enzyme mutation is the current classification system for GBM, as outlined by the World Health Organization (WHO) [8]. In the Krebs cycle of glucose metabolism, IDH is an important rate-limiting enzyme. In the context of cancer, mutations in IDH have been associated with high levels of hypoxia-inducible factor-1α (HIF-1α) and vascular endothelial growth factor (VEGF) which promote tumor progression and metastasis, whereas high levels of 2-hydroxyglutaric acid (2-HG) inhibit stem cell differentiation [9]. Recent literature also suggests that Nuclear Factor Erythroid 2-Related Factor 2 (Nrf2) transcription factor plays a role in the pathogenesis and progression of gliomas with IDH mutations [10].

The primary role of Nrf2 in normal cells is to regulate the protective response against oxidative stress.  Nrf2 is not active in normal oxygenated environments. However, Nrf2 migrates to the nucleus in response to oxidative stress and activates a number of antioxidative enzymes, neutralizing reactive oxygen species and ultimately promoting homeostasis of cellular mechanisms [11]. Nrf2 can also respond to oxidative stress through the production of NADPH by promoting the expression of NADPH-producing enzymes, such as IDH and other glucose metabolism regulators [12]. Recent studies have demonstrated that Nrf2 levels are significantly higher in GBM cell lines, demonstrating that Nrf2 expression and IDH mutations are implicated in the molecular pathogenesis of gliomas [11]. Immunohistochemical analysis of high-grade GBM tumors also revealed higher Nrf2 expression which was additionally confirmed by RNA expression analysis of publicly available tumor databases [13]. As a result, Nrf2 could be contributing to activation of other mechanisms associated with transition to a more aggressive mesenchymal tumor subtype [14].

First-line clinical treatment of GBM remains surgical resection followed by radiation therapy [15], despite studies suggesting that tumors tend to have heterogeneous environments [16, 17]. Hence, there is a need to provide targeted therapy for patients. Currently, it is recommended to adapt treatment based on the status of IDH mutation in addition to the presence of *O*6-methylguanine-DNA methyltransferase (MGMT) promoter methylation which has shown to improve efficacy [15]. Thus, the latest advances in molecular research  of pathogenic GBM pathways are of particular importance for the development of new clinical approaches. Nrf2 transcription factor could be a major effector in GBM progression due to its involvement in multiple pathogenic pathways (Fig. 1). This review is aimed to summarize current understanding of the Nrf2 role of GBM molecular signaling and present potential therapeutic approaches.

## Role of Nrf2 in Glioblastoma

Nrf2 is known to be a transcription factor with specific importance in cancer therapy. It is involved in a variety of different mechanistic cascades responsible for anti-metabolic, antioxidative, anti-inflammatory, and anticancer effects [18]. Its role in cancer has made it a potential therapeutic target that deserves closer attention.

**Fig. 2:**
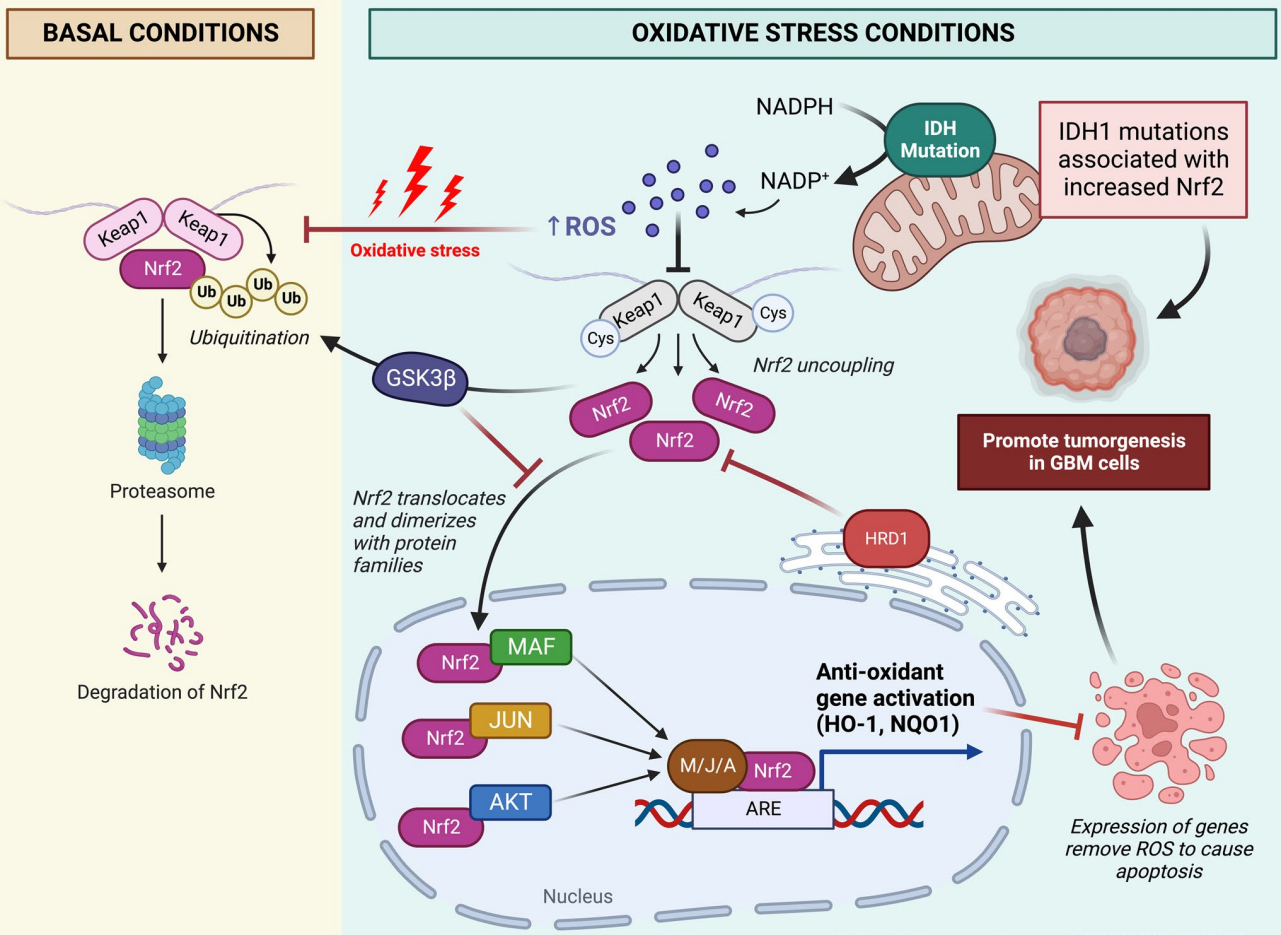
A general overview of the Nrf2 pathway in basal conditions vs. oxidative stress and how it influences the pathogenesis of GBM. (Created with biorender.com)

Tonelli et al. described Nrf2 based regulation, clearly delineating the mechanistic roles of Nrf2 in multiple cellular processes such as autophagy, cell quiescence, as well as its important DNA-protective properties achieved through regulation of antioxidant responses. Under normoxic conditions, Nrf2 is bound to ubiquitin substrate adaptor Keap1, a structural component of the E3-ubiquitin ligase complex, which maintains its constant ubiquitination and subsequent degradation. However, during a state of stress, reactive oxygen species oxidize the cysteine residues on Keap1, leading to its uncoupling from Nfr2. This allows the Nrf2 molecule to migrate to the nucleus where it binds to the Maf protein and creates the Nfr2–Maf complex. This dimeric complex binds to antioxidant response elements (ARE) which regulate transcription of proteins involved in antioxidation, detoxification and metabolism. Any mutations to the Nfr2 or Keap1 related genes would therefore significantly impact the protective role of Nfr2 against stressors and increase the risk for neoplastic development [19]. This process is depicted in Fig. 2.

GBM research has continued to further implicate Nrf2 as an important player in disease development. A study by Zhu et al. [20] analyzed the expression of Nrf2 in GBM stem cells based on its respective marker, CD133. After isolating GBM cells based on this marker, the study found Nrf2 to be overexpressed in CD133+ GBM stem cells in comparison with CD133− stem cells, indicating that Nrf-2 expression could be contributing to the malignant proliferation and differentiation of GBM stem cells [20]. A study by Haapasalo et al. [13] looked at Nrf-2, DJ1 and SNRX1 in astrocytic gliomas ranging from human grades II–IV. They identified that Nrf-2 was highly expressed in high malignancy grade glioma cells and noticed that IDH 1 mutations were associated with increased Nrf-2 expression [13]. Ahmad et al. [21] published a study that investigated the relationship between the activation of telomerase and metabolic dysregulation in the progression of glioma using Costunolide, a human-telomerase reverse transcriptase (HTERT) pharmacological agent. They found that Costunolide-treated tumors had decreased Nrf2 expression, while ectopic Nrf2 expression led to a decrease in Costunolide-induced ROS generation [21]. It was shown that while TERT knockdown models were found to exhibit reduced Nrf-2 levels, an overexpression of Nrf-2 was associated with increased TERT expression. As a result, the study suggests that the Nrf2-TERT loop is implicated as a defense mechanism against oxidative stress in glioma cells [21]. Pan et al. [22] recognized a physiologic anti-apoptotic role of Nrf2. and sought to elucidate whether a similar activity would be observed in human GBM U251 cell line. its specific role in glioma cells which they investigated using the human GBM cell U251. In their study, plasmids were transfected to either up- or downregulate Nrf-2 expression and apoptosis rates were measured along with several other pro-apoptotic factors such as caspase 3, 9 and Bcl-2 [22]. Their work established that down regulating Nrf-2 led to an enhancement of apoptotic rates and apoptosis-associated factors (i.e., caspases, Bcl-2, HO-1, etc.) thus implicating that Nrf-2 participates in apoptotic regulation in glioblastoma cells [22]. Considering these findings together, it can be concluded that in addition to its antioxidative function, Nrf-2 is an important regulator of GBM cell differentiation, proliferation, and survival.

## The interplay of Nrf2 with other molecular signaling pathways in GBM

The Nrf2 pathways regulate the defense mechanism against endogenous and environmental stimuli that lead to oxidative and electrophilic stress (Table 1). It up-regulates various ARE-containing genes such as NAD(P)H quinone oxidoreductase-1 (NQO1) and heme oxygenase-1 (HO-1) to protect the body against stress [23]. It modulates cell metabolism to enhance the antioxidant effect through a variety of mechanisms such as the pentose phosphate pathway and fatty acid oxidation while down regulating lipid metabolism [24]. However, it has been found that Nrf2 promotes cell proliferation of glial cells and causes tumor growth [25]. Various studies have found that different molecular signaling pathways are implicated in the pathogenesis of glioma, with the transforming growth factor beta (TGFβ) pathway being the most prominent [26]. TGFβ is an oncogenic factor in GBM which regulates the activity of platelet-derived growth factor B (PDGFB). High levels of PDGF proteins are associated with poor prognosis in glioma patients. PDGFB specifically has been found to induce cell proliferation, renewal, angiogenesis, and subsequently promote cell survival [28–29]. Evidence from patient-derived GBM stem cell experiments suggests that TGFβ upregulates the expression of NADPH oxidase 4 (NOX4) protein, which also occurs in other cancer cell types like hepatocellular carcinoma [30]. Similarly, higher NOX4 expression was correlated with a worse prognosis in patients with GBM [31]. An investigation done by Kim et al. has shown that Nrf2 inhibition can suppress the angiogenesis and growth of colon tumors through the inhibitory effects on Hypoxia-induced factor-1 (HIF-1) alpha activation [32]. Levels of HIF-1 alpha rapidly increase under hypoxic conditions. Pathways upregulated by HIF-1 alpha induce expression of multiple genes which govern cellular adaptations to hypoxia and also affect other processes such as chemotaxis, cell proliferation, survival and migration through the extracellular matrix [33]. Under non-oncogenic conditions, oxygen-induced HIF-1 alpha proteins are rapidly degraded through hydroxylation of proline residues by specific enzymes, such prolyl hydroxylase domain proteins (PHDs). Hydroxylation promotes the complexing of HIF-1 alpha with the Von Hippel–Lindau (VHL) protein, a subunit of the E3 ubiquitin ligase, followed by proteasomal degradation [33, 34]. Choi et al. have demonstrated that the Nrf2 downstream target, heme oxygenase-1 (HO-1), has the capability to stabilize HIF-1 alpha and promote cancer cells survival even in low oxygen environments [35]. This finding was confirmed in another study showing the correlation between Nrf2 and HIF-1 alpha levels with high Nrf2 levels being associated with poorer GBM outcomes [36].

**Table 1:** A summary of the interactions between Nrf2 and other molecular signals implicated in GBM

Molecular pathway	Interplay with Nrf2 signal
TGFβ pathway	The TGFβ pathway induces the action of PDGFB; now, this PDGFB further up-regulates a secondary cytokine, LIF, by promoting tumor cell migration. Nrf2 has also been found to induce PDGFB
HIF-1 alpha	Nrf2 can suppress angiogenesis and growth of colon tumors through the inhibition of HIF-1 alpha activation
ERK	ERK have been found to be hyperactivated in human GBM which induces the activation and expression of Nrf2
PI3K	PI3K inhibitor can decrease the expression levels of the following proteins in A549 cell lines: Nrf2, HO-1 and NQO-1

Nrf2 expression has additionally been shown to be dependent on other important GBM-associated cell proliferation pathways regulated by extracellular signal-regulated kinases (ERK) and phosphoinositide 3-kinase (PI3K) [37]. Chowdhry et al. have demonstrated that the PI3K inhibitor decreases the levels of Nrf2, HO-1 and NQO-1 in A549 lung carcinoma cell lines [38]. Although these in-vitro studies suggest that the ERK and PI3K pathways regulate the expression of Nrf2, the details of the regulatory pathway and additional contributing molecular mechanisms are still under investigation.

## DNA modification epigenetic mechanisms and Nrf2 activity

The role of epigenetics in GBM has been drawing much attention due to its implication in resistance to current therapies [39]. Temozolomide (TMZ) is an alkylating agent recommended in combination with radiation to treat GBM [15]. TMZ exhibits cytotoxicity through methylation of guanine and adenine DNA residues resulting in the DNA replication errors and leading to cell cycle arrest followed by apoptosis. This activation of the DNA repair system is essential for TMZ susceptibility [40]. Methylguanine methyltransferase (MGMT) reverses TMZ-induced methylation leading to resistance [41]. Suppression of MGMT expression by promoter methylation has been associated with better prognosis and TMZ susceptibility, however, a subset of patients is still resistant to alkylating agents [42]. It is important to point out that MGMT methylation pattern has not been shown to evolve with the tumor progression. Brandes et al. monitored MGMT promoter methylation state throughout the disease and observed stable patterns for most patients [40].

Currently, there are no studies specifically elucidating epigenetic regulation of Nrf2 in GBM. However, further research in this area could be promising for new therapeutic approaches as evidence is emerging that epigenetic mechanisms could be involved in Nrf2 regulation in other cancer types. DNA modifications such as methylation, acetylation, chromatin remodeling, and non-coding RNA regulation have been seen to involve the regulation of major metabolic pathways contributing to cancer progression [43]. One of the first studies to analyze the epigenetic underpinnings of Nrf2 has been found that the first 5 CpG sites of Nrf2 genetic sequence were hypermethylated in 96% of the analyzed prostate tumors compared to normal prostate tissues, suggesting the importance of methylation in the expression of Nrf2 [44]. Additionally, Kozono et al. described an important action of lysine-specific demethylase 1 (LSD1) in suppressing overexpression of the oncogenic c-MYC transcription factor through preventing histone trimethylation [45]. This may tie in with the Nrf2 regulation as studies have observed the role of c-MYC in the activation of Nrf2, leading to malignant progression in head and neck squamous carcinoma cell lines [46]. In vitro studies on gliomas also demonstrated Nrf2 levels to be correlated with c-MYC expression but the exact mechanism is widely unknown [47].

## Role of miRNA NRF2 regulation

MicroRNAs (miRNA) are short noncoding RNA strands involved in the regulation of gene expression by binding to specific mRNA molecules [48]. The first miRNA to be implicated in Nrf2 regulation was miR-144, as the expression of this molecule inhibited Nrf2 [49]. Other forms of cancer have shown an involvement of miRNA, and subsequent downregulation of Nrf2 which may potentially be involved with the cancer’s underlying mechanism. For example, breast cancer cells were shown to express a variety of miRNA such as miR-27a, miR142-5p, miR-144 and miR-153, and these cells showed a suppression of Nrf-2 mRNA and protein levels [50]. Similarly, the role of microRNAs has been studied in GBM. One study showed that up-regulated miRNAs including miRNA-323, miRNA-210, and miRNA-155 could be associated with improved mortality in GBM patients [51]. Another study by Wang et al. [52] found that changing levels of miRNA-128 and miRNA-342 could have implications towards the histopathological grading of GBM [53]. In their review article, Saadatpour et al. identify a number of miRNA that have been shown to have altered levels in various glial cell models, thus further establishing the role of miRNA in the pathogenesis and progression of GBM [52]. These miRNA include miR-221, miR-204 and miR-16 among the up-regulated microRNA while miR-195, miR-633 and miR-136 among the down regulated microRNA in GBM. However, of note, PubMed was unable to yield results for “miRNA, Nrf-2 AND glioblastoma multiforme”, suggesting that further research must be conducted to target the activity of miRNA and their relationship to Nrf2 in the context of GBM. This could implicate a potential therapeutic target for GBM management.

## Emerging role of NRF2 in glioblastoma phenotype switch and treatment resistance

Highly aggressive course of glioblastoma tumors has been suggested to result from their heterogeneity. Through clinical experience and comprehensive molecular analysis of tumors, clinically relevant subtypes of GBM with similar gene expression and methylation profiles have been determined [54, 55]. A broadly accepted classification of glioblastoma tumors includes proneural, neural, classical and mesenchymal subtypes. At the time of this classification, the authors considered transition between subtypes to be unlikely [55]. Subsequent investigation has determined that glioblastoma tumors exhibit heterogeneity of different cell subtypes with each sample possessing populations of cells with multidrug and radiotherapy resistance. Multidrug-resistant phenotypes have been associated with mesenchymal profile [39]. This subtype of GBM cells is highly invasive [54] and typically expresses neural stem cell markers [56]. Gene enrichment analysis of these tumors revealed upregulation of the pathways associated with mesenchymal transition, extracellular matrix (ECM) receptor interactions, antigen processing and presentation, ATP-binding cassette (ABC) transporters, and drug metabolism [39]. Those features of cancer cells are crucial for their survival and resistance [54]. Interestingly, some treatment strategies, such as radiation and chemotherapeutic approaches, could induce transition to mesenchymal phenotype [56].

An important role of Nrf2 in mesenchymal transition was demonstrated in the 2019 study by Polonen et al. Based on microarray and RNA-seq gene expression analysis of GBM samples, patients whose tumors had high levels of Nrf2 survived shorter time periods. In addition, Nrf2 activity was found to be correlated with tumor grade with none of the patients with grade I tumors having high levels of Nrf2. Nrf2 activation was linked to dysregulation of autophagy. Specifically, the authors identified co-regulatory positive feedback between autophagy regulating protein complex Sequestosome1 (SQSTM1/p62) and Nrf2 which could promote mesenchymal phenotype. It has been suggested that Nrf2 could directly promote expression of Slug and ß-catenin mesenchymal markers by interaction with their enhancer DNA region [14]. A different study confirmed these findings by implicating mesenchymal transition with TLR4–p38–Nrf2 pathway-mediated p62 overexpression [57].

Nrf2 has also been suggested to potentiate GBM resistance to redox antitumor agents [58]. Cannabidiol (CBD) is a non-toxic and non-psychoactive redox modulator used to induce cytotoxic reactive oxygen species (ROS) in GBM cell lines. However, cells gained resistance to CBD through activation of NRF2-mediated antioxidant response. Resistant cells were found to express mesenchymal markers (CD44, TNSFR10, CEBPB). The authors concluded that GBM resistance to CBD was due to Nrf2-mediated antioxidant response and an adaptive reprogramming toward mesenchymal phenotype [58].

## Significance of NRF2 in clinical practice and GBM treatment strategies

**Fig. 3:**
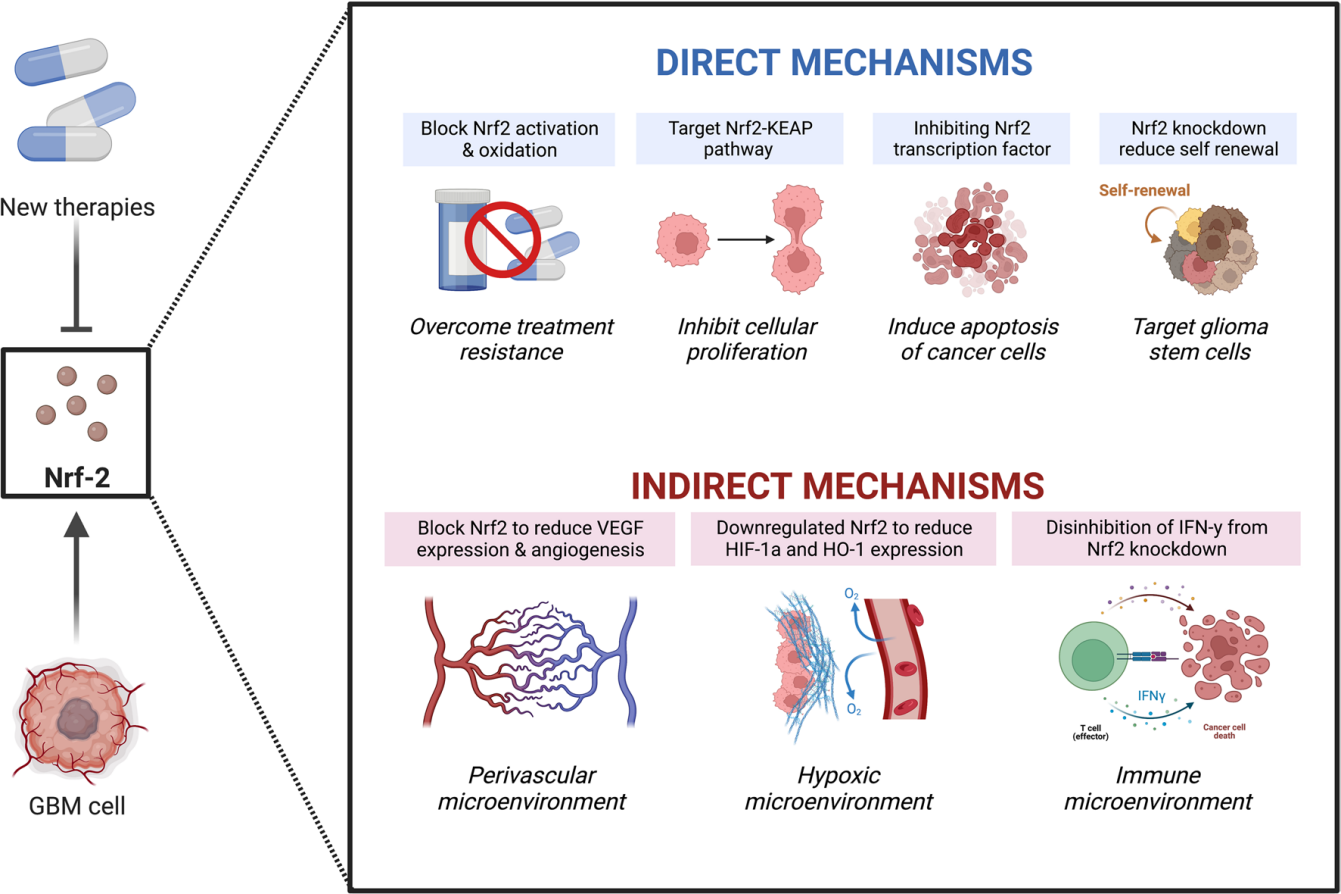
Schematic showing potential targets for GBM therapy related to Nrf2 signaling and expression. (Created with BioRender.com)

Despite development of new therapeutic approaches in treatment of solid tumors, the survival prognosis of GBM patients remains poor. Due to the extensive role of Nrf2 in cell regulation, its associated pathways have become potential GBM therapeutic targets (Fig. 3). Nrf2–Keap1 signaling has been shown to be essential for high grade tumor development and progression [11]. An increased level of Nrf2 expression in glioblastoma was found to be protective against antitumor therapies, while blocking Nrf2 signaling could inhibit the disease progression [59]. NRF2 and related genes were found to be negatively correlated with glioblastoma patient survival rates [60]. In high grade gliomas, cytoplasmic NRF2 expression is associated with poor prognosis [13]. NRF2 target genes were found to be elevated in 32.7% of glioblastomas which could not be explained by the dysregulation of KEAP1 pathway [10] suggesting multiple NRF2-mediated mechanisms contributing to the disease. The need for simultaneous targeting of multiple pathways, with no demonstrated success of a single therapy approach, presents a challenge in finding an effective therapeutic regimen. Currently, direct, and indirect approaches of Nrf2 targeting are considered in GBM treatment [59], however, clinical trials still have not identified a therapy that would significantly improve patients’ outcomes. Nonetheless, Nrf2 associated pathways remain an attractive therapeutic target with a number of potential drug-candidates being tested in-vitro. We summarized recent investigative efforts in GBM treatment strategies and emphasized a promising therapeutic value of Nrf2 inhibition.

1. Direct Nrf2 targeting approaches:
  By these mechanisms, Nrf2-mediated molecular pathways are targeted to overcome treatment-resistance, inhibit GBM cells proliferation, invasion, migration, and promote apoptosis [59]. Nrf2 targeting could also reduce the expression of self-renewal-related factors, such as Bmi1, Sox2 and cyclin E, and inhibit oncogenic stem cells proliferation [59, 61].
  - Overcoming resistance and apoptosis induction:
    Resistance to both, radiotherapy, and chemotherapy, remains the biggest challenge in glioblastoma treatment. Nrf2 regulates the expression of antioxidant and phase II drug-metabolizing enzymes, and acts as a protective factor for tumor cells against environmental oxidative stressors as well as xenobiotics such as chemotherapeutic drugs and ionizing radiation [63–64]. Downregulation of Nrf2 pathways could lead to decrease in the antioxidant response and ultimately make GBM cells more susceptible to the environmental stressors. In tumors with IDH1 mutation, Nrf2 plays a protective role by prompting glutathione (GSH) synthesis and reactive oxygen species scavenging. Pharmacologic inhibition of the Nrf2/GSH pathway via brusatol administration exhibited a potent tumor suppressive effect in-vitro and in-vivo [63]. Therefore, Nrf2 knockdown could make glioma cells more susceptible to chemotherapy and bypass resistance to conventional therapies. Temozolomide (TMZ) is currently a gold standard in GBM chemotherapeutic approaches. However, it has been shown that TMZ combined with radiation could induce acquired Nrf2-mediated resistance. Interestingly, subsequent Nrf2 inhibition could restore therapeutic response [37]. Sphingosine-1-phosphate analogue has been shown to reduce expression of Nrf2 and its target proteins in human glioblastoma cell lines, making cells more susceptible to TMZ [65]. As such, inhibition of Nrf2 activity could be a potential clinical approach to overcome treatment resistance [66, 67].
    Major contributors to the GBM resistance against chemotherapy and radiotherapy are altered cell-cycle regulatory pathways that prevent autophagy and apoptosis [68]. There is evidence that Nrf2 can block apoptotic death of malignant cancer cells [69]. Inhibition of Nrf2 could make cancer cells more susceptible to apoptosis [70]. Protein disulfide isomerase (PDI), commonly overexpressed in glioblastoma, has been suggested to modulate apoptotic signaling and was found to regulate the Keap1/Nrf2 system and redox balance. It was discovered that PDI inhibition impairs Keap1/Nrf2 signaling with subsequent apoptosis induction [71]. A PDI inhibitor, pyrimidotriazinedione 35G8, was found to be toxic in human glioblastoma cell lines. Ferroptosis is an iron-dependent programmed cell death that could also be dependent on Keap1/Nrf2 signaling. Interestingly, 35G8-induced cell death did not proceed via apoptosis or necrosis, but by a mixture of autophagy and ferroptosis [68]. Corilagin is another compound that is capable of downregulating NRF2 expression, and has been shown to induce apoptosis and autophagy [69]. Valproic acid, melatonin, and all-trans retinoic acid are all capable of apoptosis induction by inhibiting the Nrf2-ARE signaling in TMZ-resistant cell lines and are believed to act as chemotherapeutic sensitizers in the treatment of chemotherapy resistant glioblastoma [70, 72]. Additionally, melatonin was found to antagonize hypoxia-mediated GBM cell migration and invasion via inhibition of HIF-1α [73].
  - Inhibition of regulatory pathways and cell proliferation:
    Glioblastoma cells have a high proliferation rate with the Nrf2–Keap1 pathway acting as a switch to the malignant cell signaling, promoting metabolic changes, cell proliferation and survival [11]. Multiple compounds have been tested for clinical efficacy, however, none of the proposed approaches has demonstrated success in clinical trials. A natural product obtusaquinone (OBT) has been shown to downregulate the Nrf2 pathway through binding to Keap1 cysteine residues and promoting its proteasomal degradation [74].
    Malignant GBM phenotype is dependent on metabolic alterations which also show an association with the Nrf2 activity. Aggressive glioblastoma cell lines exhibit preference for glutamine over glucose as an energy source. Transition to glutaminolysis has been shown to be mediated by AMP-activated protein kinase (AMPK) through Nrf2 pathways activation. This makes the AMPK–Nrf2 axis another potential target. Treatment with the glutaminase inhibitor, CB839, and cystine transporter inhibitor, sulfasalazine, has been able to achieve cytotoxicity through inducing glutamine starvation [68].
    Alternative method of Nrf2 pathways targeting is interfering with the cell signaling mediated by tyrosine kinases. Chrysin**,** an active natural bioflavonoid, suppresses proliferation, migration, and invasion in glioblastoma cell lines via ERK/Nrf2 signaling pathway [75]. Inhibitor of PI3K, buparlisib, has shown antitumor activity in glioma models, however subsequently failed to demonstrate sufficient therapeutic efficiency in clinical trials [76]. Imatinib, a protein kinase inhibitor commonly used in leukemia treatments, also showed no measurable activity in patients with newly diagnosed or recurrent glioblastoma [77]. Synergistic approaches have shown to be more promising. Stage II clinical trial determined perifosine, an inhibitor of PI3/AKT pathway, to be not effective GBM treatment as a single therapy, however demonstrated an antitumor effect as a combined treatment with an mTOR inhibitor temsirolimus [78].
2. Indirect Nrf2 targeting approaches:
  Indirect treatment approaches are based on the interference with tumor microenvironment which could potentially be effective as a single therapy, or increase therapeutic efficacy of direct antineoplastic agents [59].

Glioblastoma tumors are highly vascular, which contributes to their aggressive, highly proliferative phenotype and poor patients prognosis [79, 80]. Several proangiogenic proteins have been found to be up-regulated in glioblastoma and their associations with the Nrf2 activity are being explored [81, 82]. Angiogenesis is regulated by the interplay between VEGF and Nrf2 [83]. Nrf2-induced rise in VEGF expression promotes vascular endothelial growth and tumor progression [84]. Therefore, targeting Nrf2 expression and its associated pathways could reduce angiogenesis and inhibit tumor growth.

Even though an angiogenesis-targeted approach is believed to be promising, clinical trials still have not identified a successful drug candidate. Cilengitide is a small molecule compound capable of inhibiting angiogenesis and promoting tumor cell apoptosis. Unfortunately, clinical trials have shown that it did not improve patients’ outcomes [85]. Anti-VEGF monoclonal antibody, Bevacizumab, also did not confer a significant survival advantage in patients with progressive glioblastoma [86]. Dovitinib, a potent VEGFR inhibitor, was also not found to be efficacious in Phase II clinical trials [87]. Isolinderalactone has demonstrated effectiveness in inhibiting VEGF secretion in cell culture and reduced tumor burden in mouse GBM xenograft models, however, has not yet been advanced to the clinical trials [88].

The glioma microenvironment is associated with altered immune responses promoting cancer cell survival [89]. Immunotherapy is regarded as a promising approach in GBM treatment. Regression of intracranial and spinal tumors was observed in a patient treated with pre-engineered interleukin-13 targeting T-cells [22]. Immune checkpoints inhibitors such as PD-1, PD-L1, and CTLA-4 have been shown to promote increased immune activation with the potential in GBM treatment, however, could not be progressed to the clinical trials due to associated toxicity [19]. Nrf2 is a critical regulator of the innate immune response capable of suppressing interferon-γ (IFN-γ) activity [90] and contributing to the ability of tumors to evade an immune response. Thus, inhibition of Nrf2 pathways may be an alternative way to promote anti-GBM immunity and add an additional therapeutic value.

## Conclusions

Glioblastoma is a primary brain tumor with a withering prognosis despite ongoing advances in the treatments of oncological malignancies. Several studies have associated Nrf2 overexpression with a highly malignant glioblastoma phenotype. Nrf2 is an important regulatory molecule that allows GBM tumors to maintain low immunogenicity and antiapoptotic proliferative phenotypic features. Nrf2 exhibits complex interactions with multiple cellular pathways essential for homeostasis maintenance. Nrf2 activity has been implicated in highly vascular glioblastoma phenotype and treatment resistance. In the current scientific literature, there is a consensus that Nrf2 plays an essential role in GBM pathogenesis and could be an attractive therapeutic target. Despite no demonstrated efficacy of currently proposed compounds in clinical trials, combination therapies aimed at multiple Nrf2-mediated pathways are believed to be promising. GBM-associated Nrf2-dependent molecular interactions are still being actively investigated and a number of new drug-candidate compounds have been identified.

## Data Availability

No new data generated. All the data is available in the manuscript.
